# Supplementary figures and images for: SPIN-CGNN: Improved fixed backbone protein design with contact map-based graph construction and contact graph neural network
Source: PLoS Comput Biol. 2023 Dec 7;19(12):e1011330. doi: 10.1371/journal.pcbi.1011330 (PMC10729952; doi:10.1371/journal.pcbi.1011330)

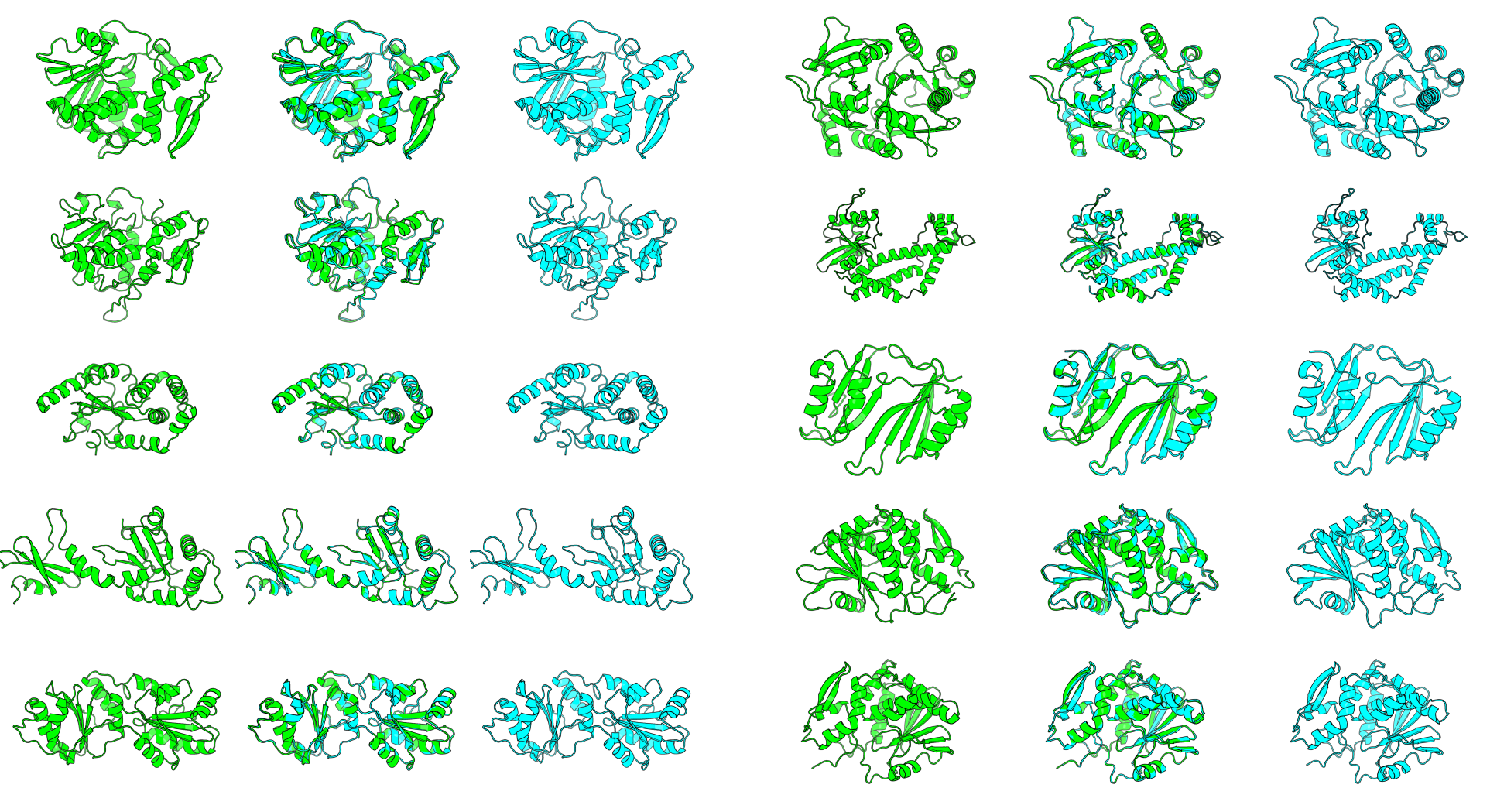

Supplement: S1 Fig — The alignments of structure pairs were presented between two test structures in green and cyan. (TIF) [file pcbi.1011330.s001.tif]

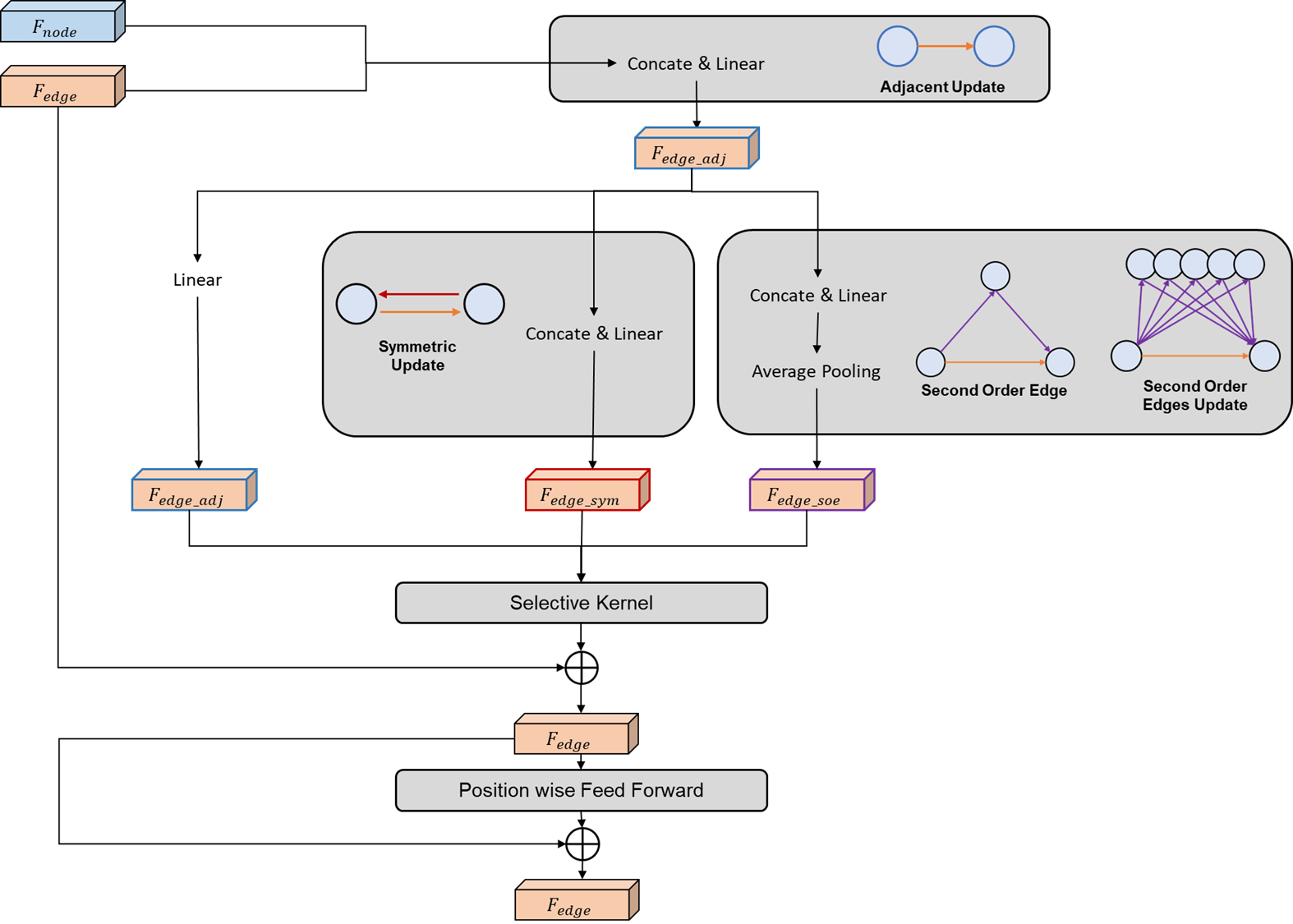

Supplement: S2 Fig — (TIF) [file pcbi.1011330.s002.tif]

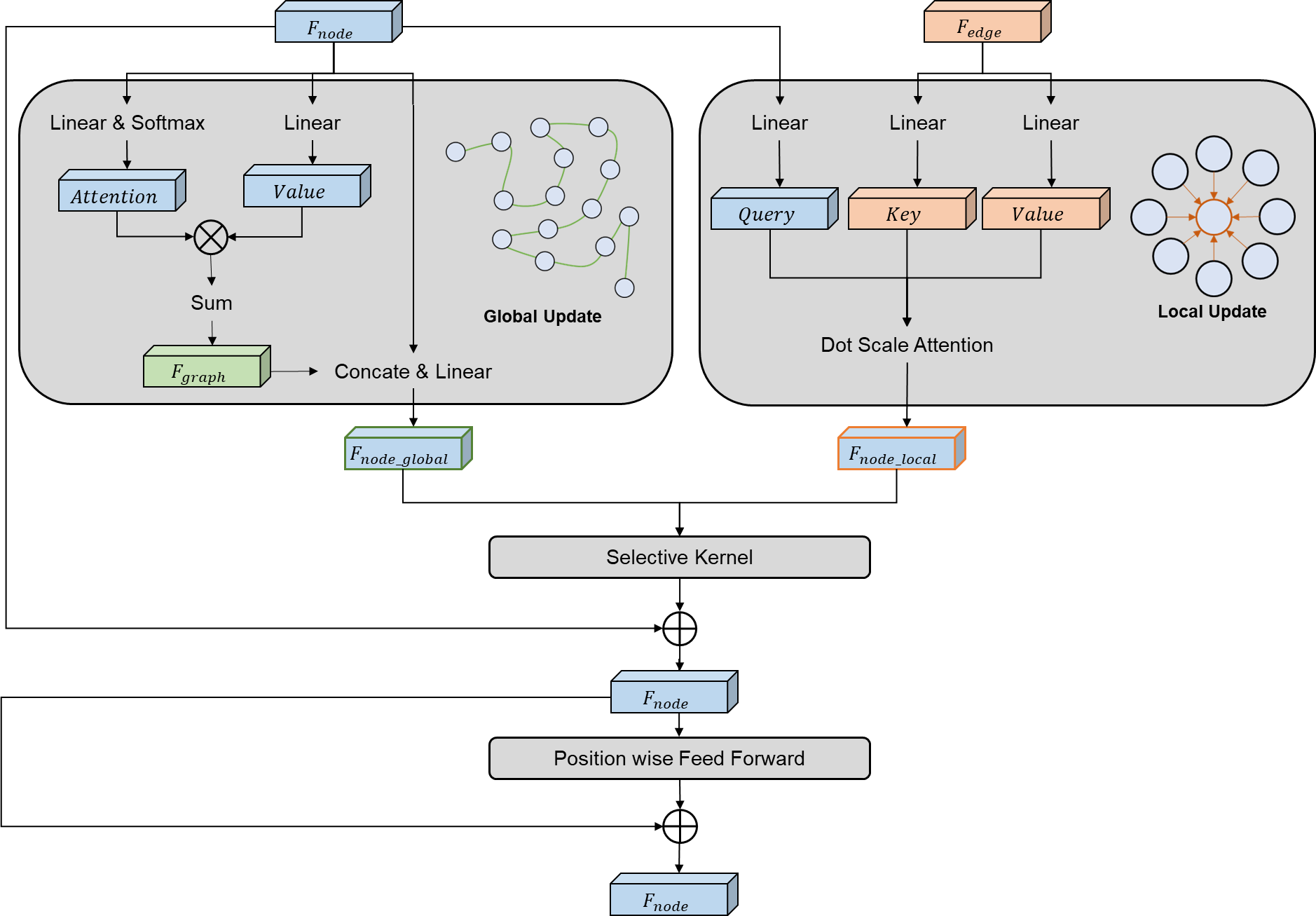

Supplement: S3 Fig — (TIF) [file pcbi.1011330.s003.tif]

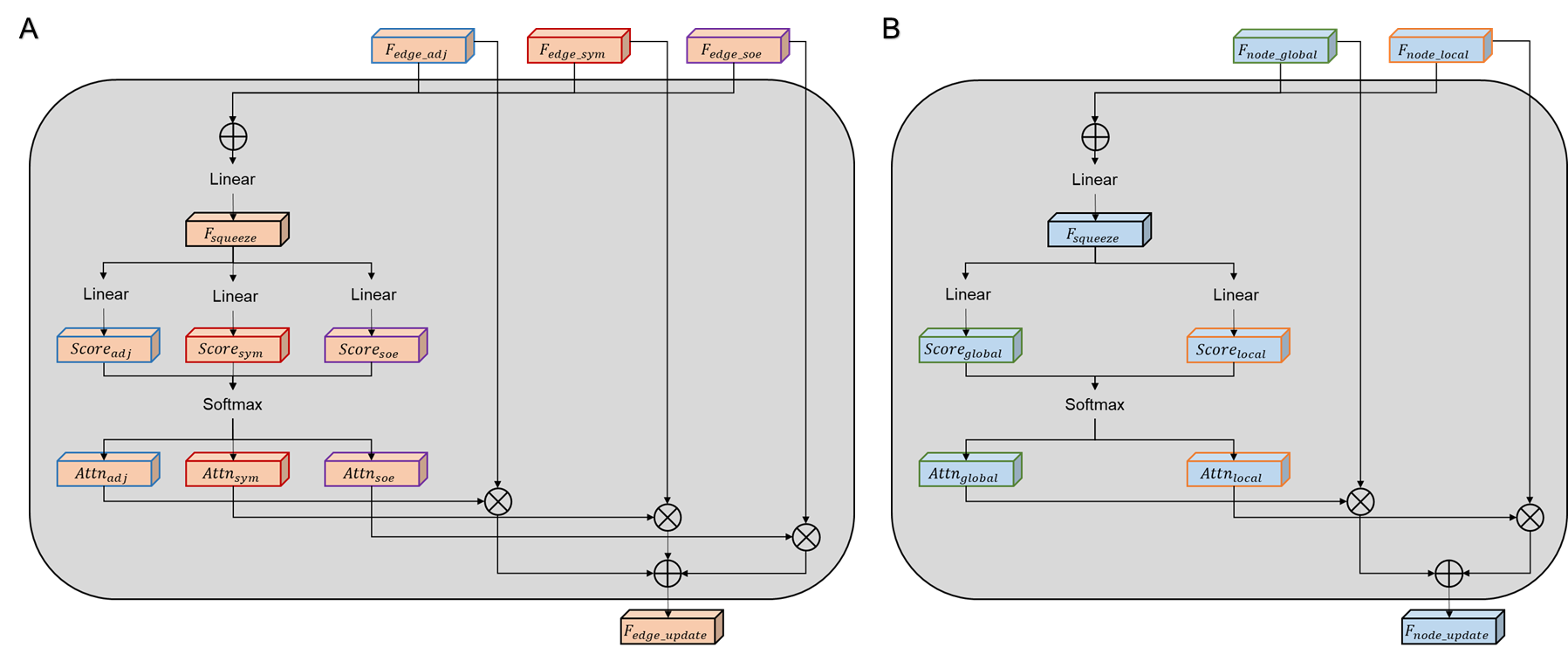

Supplement: S4 Fig — Selective kernels for edge update (A) and node update (B) in the CGNN block. (TIF) [file pcbi.1011330.s004.tif]

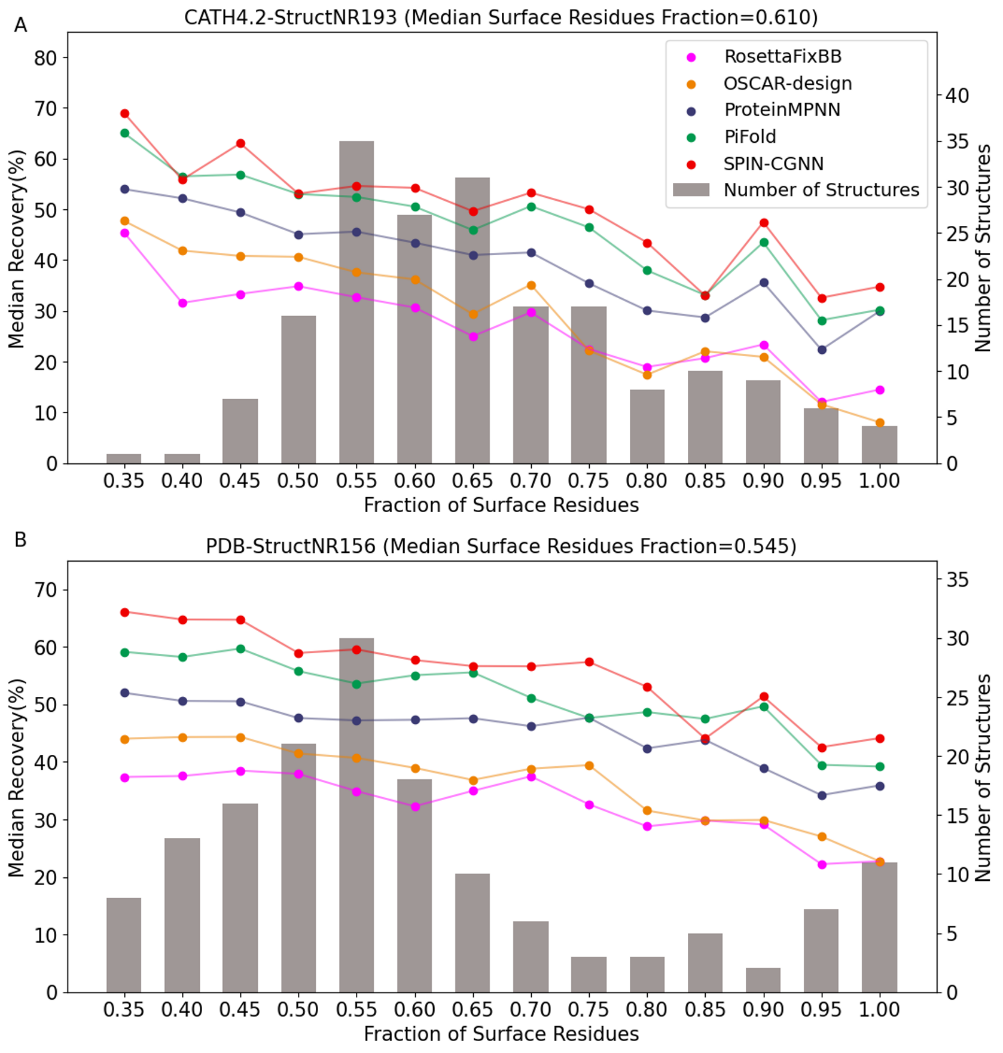

Supplement: S5 Fig — The median sequence recovery of protein targets as a function of the fraction of surface residues on CATH4.2-StructNR193 (A) and PDB-Struct156 (B) test sets given by SPIN-CGNN, in comparison with a number of other methods as labeled. Nearly identical dependence on fraction of surface residues by SPIN-CGNN for two different test sets indicates the robustness of the methods for different datasets. (TIF) [file pcbi.1011330.s005.tif]

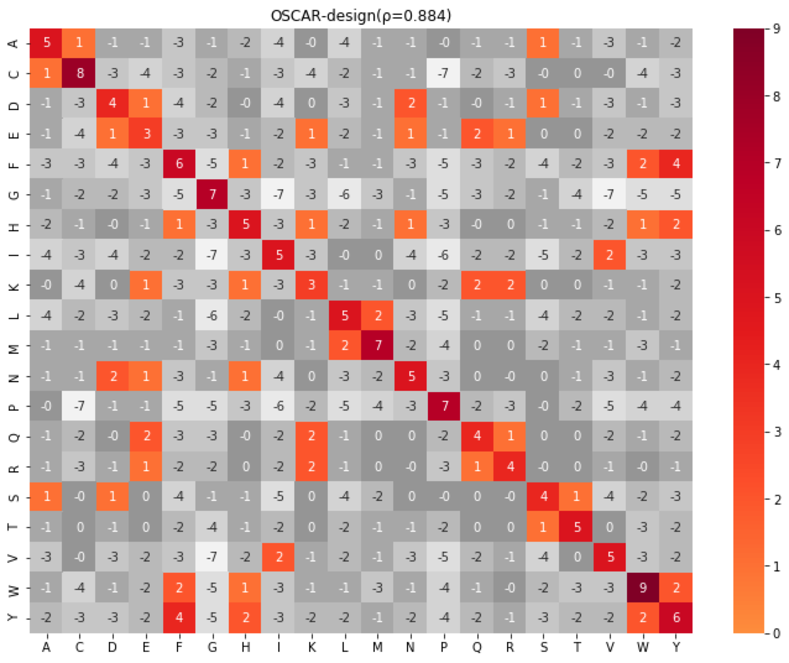

Supplement: S6 Fig — (TIF) [file pcbi.1011330.s006.tif]

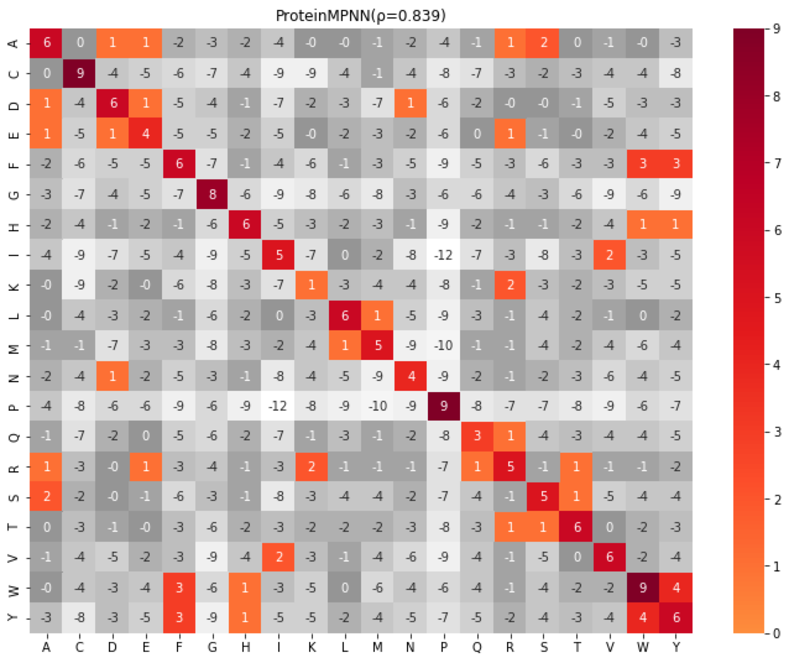

Supplement: S7 Fig — (TIF) [file pcbi.1011330.s007.tif]

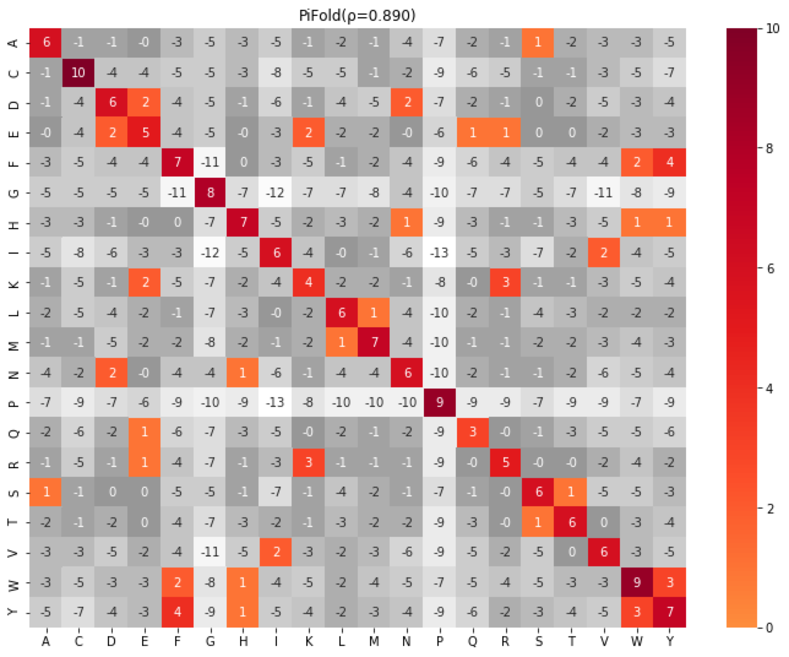

Supplement: S8 Fig — (TIF) [file pcbi.1011330.s008.tif]

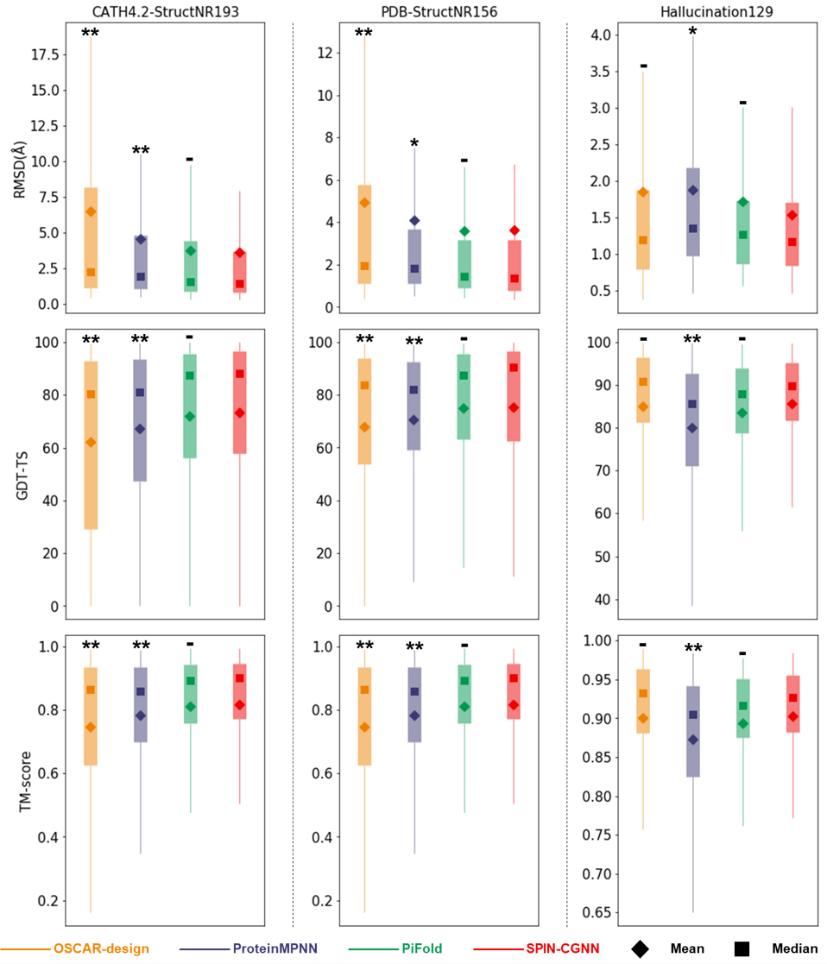

Supplement: S9 Fig — Deviations of the structures of designed sequences predicted by AlphaFold2 with PDB templates from their respective target structures on four separate test sets from left to right panels (CATH4.2-StructNR193, PDB-StructNR156, Hallucination129, and Diffusion100 test set) evaluated according to RMSD (Å), GDT-TS, and TM-score (from top to bottom panels). The statistical significance of the difference of a given method to SPIN-CGNN was marked with ‘**’ for highly statistically significant (p-value<0.01), ‘*’ for statistically significant (0.01<p-value<0.05), and ‘-’ for not statistically significant (p-value>0.05). (TIF) [file pcbi.1011330.s009.tif]

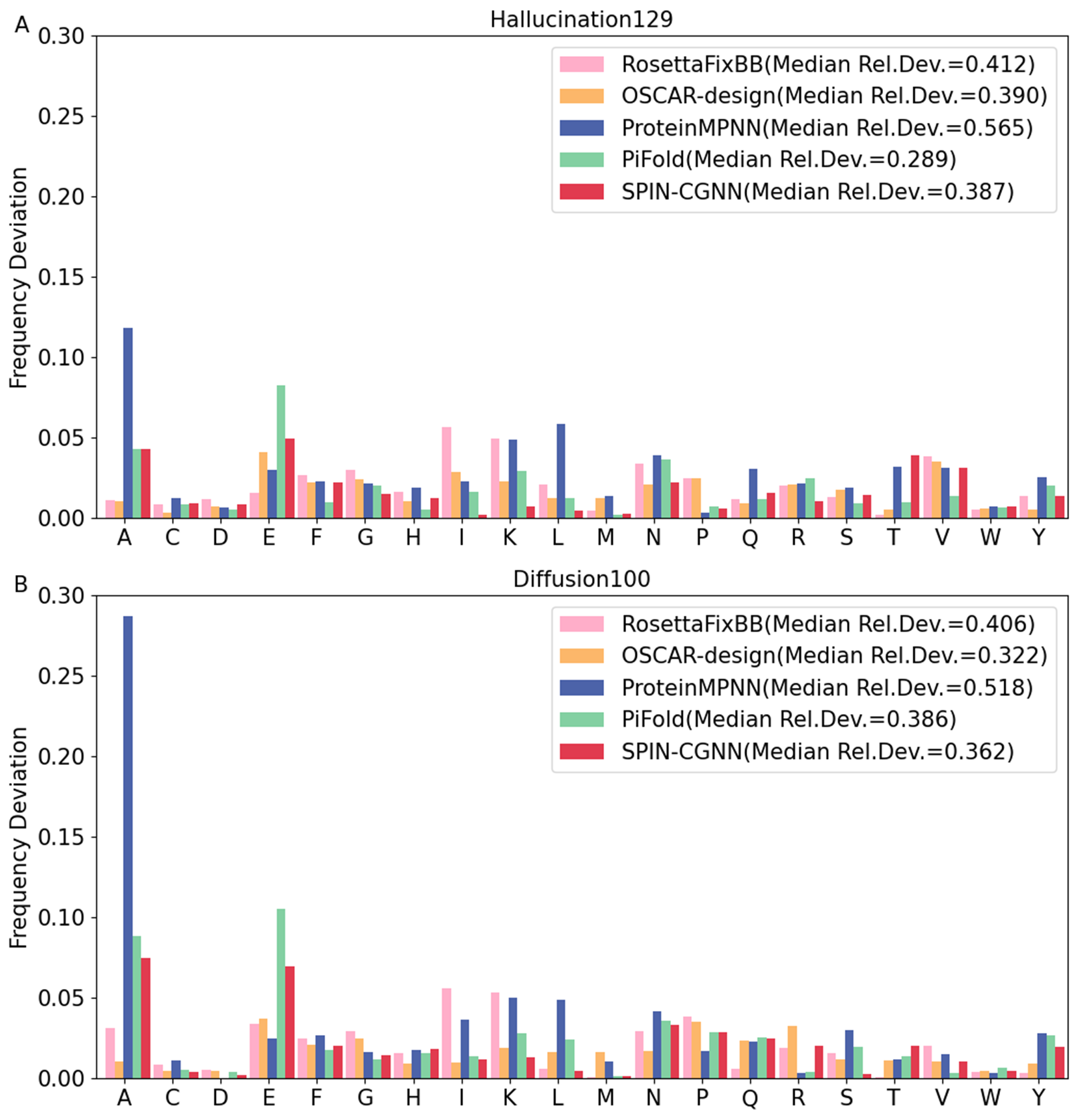

Supplement: S10 Fig — (A) Hallucination129 and (B) Diffusion100 test set. (TIF) [file pcbi.1011330.s010.tif]
