# Supplementary material for: SPIN-CGNN: Improved fixed backbone protein design with contact map-based graph construction and contact graph neural network
Source: PLoS Comput Biol. 2023 Dec 7;19(12):e1011330. doi: 10.1371/journal.pcbi.1011330 (PMC10729952; doi:10.1371/journal.pcbi.1011330)
Supplement: S1 Table — Two-layer MLP modules were employed to substitute SK modules in models without selective kernels (no-SK models). (DOCX) [file pcbi.1011330.s011.docx]

**S1 Table.** Impact of the use of selective kernels in node update and edge update according to perplexity and median sequence recovery for two test datasets (CATH4.2-StructNR193 and PDB-StructNR156). Two-layer MLP modules were employed to substitute SK modules in models without selective kernels (no-SK models).

| Model | Node SK | Edge SK | Perplexity ↓ ^a^ | Median  Recovery (%) ↑ ^a^ |
| --- | --- | --- | --- | --- |
| CATH4.2-StructNR193 | | | | |
| Model 7 | **×** | **×** | 4.49 ± 0.01 | 52.04 ± 0.33 |
| Model 8 | √ | **×** | 4.43 ± 0.02 | 52.43 ± 0.38 |
| Model 9 | **×** | √ | 4.47 ± 0.02 | 52.09 ± 0.41 |
| SPIN-CGNN | √ | √ | **4.36 ± 0.01** | **52.89 ± 0.64** |
| PDB-StructNR156 | | | | |
| Model 7 | **×** | **×** | 3.57 ± 0.00 | 57.08 ± 0.18 |
| Model 8 | √ | **×** | 3.53 ± 0.03 | 57.96 ± 0.26 |
| Model 9 | **×** | √ | 3.56 ± 0.02 | 57.61 ± 0.15 |
| SPIN-CGNN | √ | √ | **3.43 ± 0.02** | **58.54 ± 0.27** |

^a^ The average of 5 parallel tests with the standard deviations.
