# Supplementary material for: SPIN-CGNN: Improved fixed backbone protein design with contact map-based graph construction and contact graph neural network
Source: PLoS Comput Biol. 2023 Dec 7;19(12):e1011330. doi: 10.1371/journal.pcbi.1011330 (PMC10729952; doi:10.1371/journal.pcbi.1011330)
Supplement: S2 Table — (DOCX) [file pcbi.1011330.s012.docx]

**S2 Table.** Refoldability tested by AlphaFold2 with PDB templates.

| Methods | AlphaFold2 Prediction Test | | |
| --- | --- | --- | --- |
|  | Median RMSD (Å) ↓ | Median GDT-TS ↑ | Median TM-score ↑ |
| CATH4.2-StructNR193 | | | |
| Native | 1.53 | 89.41 | 0.898 |
| RosettaFixBB | 2.95 | 68.71 | 0.818 |
| OSCAR-design | 2.22 | 80.51 | 0.864 |
| ProteinMPNN | 1.94 | 81.17 | 0.859 |
| PiFold | 1.53 | 87.38 | 0.894 |
| SPIN-CGNN | **1.42** | **88.16** | **0.901** |
| PDB-StructNR156 | | | |
| Native | 1.64 | 86.93 | 0.908 |
| RosettaFixBB | 2.27 | 75.83 | 0.892 |
| OSCAR-design | 1.94 | 83.89 | 0.903 |
| ProteinMPNN | 1.82 | 82.08 | 0.908 |
| PiFold | 1.46 | 87.48 | 0.920 |
| SPIN-CGNN | **1.37** | **90.45** | **0.924** |
| Hallucination129 |  |  |  |
| RosettaFixBB | 1.39 | 86.25 | 0.913 |
| OSCAR-design | 1.19 | **90.75** | **0.932** |
| ProteinMPNN | 1.35 | 85.75 | 0.905 |
| PiFold | 1.27 | 88.00 | 0.917 |
| SPIN-CGNN | **1.17** | 89.75 | 0.927 |
| Diffusion100 |  |  |  |
| RosettaFixBB | 4.71 | 47.50 | 0.760 |
| OSCAR-design | 2.36 | 75.75 | 0.867 |
| ProteinMPNN | 3.37 | 59.63 | 0.773 |
| PiFold | 2.26 | 74.00 | 0.873 |
| SPIN-CGNN | **1.97** | **81.00** | **0.886** |
