# Supplementary material for: SPIN-CGNN: Improved fixed backbone protein design with contact map-based graph construction and contact graph neural network
Source: PLoS Comput Biol. 2023 Dec 7;19(12):e1011330. doi: 10.1371/journal.pcbi.1011330 (PMC10729952; doi:10.1371/journal.pcbi.1011330)
Supplement: S3 Table — (DOCX) [file pcbi.1011330.s013.docx]

**S3 Table.** Adding Gaussian noises to the structural coordinates slightly improved the performance of deep-learning methods (SPIN-CGNN, ProteinMPNN, and PiFold) on the Hallucination129 test set, according to the fraction of Low-Complexity Regions (LCR) and the difference between refolded and target structures in term of RMSD, GDT-TS and TM-score, except an increase of low complexity regions for SPIN-CGNN and PiFold but a reduction of LCR for ProteinMPNN.

LCR: Low-Complexity Regions. RMSD: Root Mean Square Deviation. GDT-TS: Global Distance Test-Total Score. *The TM-score of ProDesign-LE in AlphaFold2 prediction test is from ProDesign-LE paper.

| Methods | LCR (%) ↓ | AlphaFold2 Prediction Test | | |
| --- | --- | --- | --- | --- |
|  |  | Median RMSD (Å) ↓ | Median GDT-TS ↑ | Median TM-score ↑ |
| ProteinMPNN | 32.96 | 1.38 | 84.75 | 0.903 |
| ProteinMPNN(+noise) | 24.27 | 1.37 | 86.00 | 0.903 |
| PiFold | 17.19 | 1.26 | 88.25 | 0.916 |
| PiFold(+noise) | 20.27 | 1.20 | 89.25 | 0.925 |
| SPIN-CGNN | 14.91 | 1.19 | 89.00 | 0.923 |
| SPIN-CGNN(+noise) | 26.21 | **1.13** | **90.50** | **0.928** |
