# Supplementary material for: SPIN-CGNN: Improved fixed backbone protein design with contact map-based graph construction and contact graph neural network
Source: PLoS Comput Biol. 2023 Dec 7;19(12):e1011330. doi: 10.1371/journal.pcbi.1011330 (PMC10729952; doi:10.1371/journal.pcbi.1011330)
Supplement: S4 Table — ‘**’ denoted p-value < 0.01, ‘*’ denoted 0.01 < p-value < 0.05, and ‘-’ denoted P-value > 0.05. (DOCX) [file pcbi.1011330.s014.docx]

**S4 Table.** Statistical significance between a given method to SPIN-CGNN for the structural difference between target structures and AlphaFold2-predicted structures for designed sequences according to Root Mean Square Deviation (RMSD), Global Distance Test-Total Score (GDT-TS) and TM-Score for three test sets. ‘**’ denoted p-value < 0.01, ‘*’ denoted 0.01 < p-value < 0.05, and ‘-’ denoted P-value > 0.05.

| Methods | RMSD | GDT-TS | TM-score |
| --- | --- | --- | --- |
| CATH4.2-StructNR193 | | | |
| Native | 3.29E-01  - | 1.67E-03  ** | 7.21E-05  ** |
| RosettaFixBB | 3.10E-16  ** | 3.55E-22  ** | 6.05E-18  ** |
| OSCAR-design | 4.48E-09  ** | 3.08E-10  ** | 1.64E-11  ** |
| ProteinMPNN | 2.419E-04  ** | 1.73E-08  ** | 3.66E-09  ** |
| PiFold | 3.98E-01  - | 1.02E-02  * | 2.50E-03  ** |
| PDB-StructNR156 | | | |
| Native | 7.56E-1  - | 8.83E-1  - | 9.17E-2  - |
| RosettaFixBB | 3.10E-06  ** | 3.09E-12  ** | 7.77E-07  ** |
| OSCAR-design | 1.48E-04  ** | 3.09E-06  ** | 6.75E-04  ** |
| ProteinMPNN | 5.02E-02  - | 3.64E-09  ** | 4.35E-03  ** |
| PiFold | 4.32E-01  - | 1.14E-01  - | 8.27E-01  - |
| Hallucination129 |  |  |  |
| RosettaFixBB | 1.27E-03  ** | 8.83E-06  ** | 5.11E-05  ** |
| OSCAR-design | 4.00E-01  - | 5.54E-01  - | 5.17E-01  - |
| ProteinMPNN | 5.31E-01  - | 3.26E-03  ** | 5.57E-03  ** |
| PiFold | 5.87E-01  - | 1.87E-01  - | 1.24E-01  - |
| Diffusion100 |  |  |  |
| RosettaFixBB | 1.03E-05  ** | 3.63E-09  ** | 6.70E-09  ** |
| OSCAR-design | 1.31E-01  - | 1.13E-01  - | 6.28E-02  - |
| ProteinMPNN | 3.09E-03  ** | 9.20E-07  ** | 7.18E-08  ** |
| PiFold | 8.71E-01  - | 6.76E-01  - | 4.04E-01  - |
