# Supplementary material for: SPIN-CGNN: Improved fixed backbone protein design with contact map-based graph construction and contact graph neural network
Source: PLoS Comput Biol. 2023 Dec 7;19(12):e1011330. doi: 10.1371/journal.pcbi.1011330 (PMC10729952; doi:10.1371/journal.pcbi.1011330)
Supplement: Attachment — Submitted filename: Response-to-reviewers.docx [file pcbi.1011330.s015.docx]

Response to reviewers’ comments

Reviewer 1:

1. I appreciate the effort the authors put into retraining ProteinMPNN and PiFold; however, the authors should compare the performance of these programs based on the publicly released, pretrained models by each respective lab. It is not the author’s responsibility to improve the pipeline or training procedure for the control methods. Moreover, others could argue that the results are worse due to the retraining and not because of methodological improvements by SPIN-CGNN. Thus, the results of the default models should be provided whenever possible. If redundancy between the training and test sets is an issue, the authors may examine the performance on a set of non-redundant targets.

Reply: We would like to clarify the reasoning behind our decision to compare the performance of these programs based on retrained models.

In the case of ProteinMPNN, the official-released models were trained on a large PDB dataset with Gaussian backbone coordinate noise, for designing multi-chain models. We cannot compare our method to the official-released models due to the redundancy between their training and our test sets. Their training set is unfortunately unavailable for constructing a non-redundant test set for comparison with our method. Thus, to ensure a fair comparison, we have to retrain a ProteinMPNN model on CATH4.2 training set without Gaussian backbone coordinate noise. This is a common practice as we found that PiFold also retrained a ProteinMPNN model for their method comparison.

As for PiFold, the pretrained model of PiFold is currently not available to the best of our knowledge. As a result, we faithfully retrained the ProteinMPNN and PiFold models following the instructions from their respective papers. We have included the details of our retraining process in the supplementary data for transparency.

We would like to emphasize that the performance of our retrained models is essentially the same as the models retrained in the PiFold paper. Therefore, the models retrained in this work are sufficient for our comparison purpose.

We understand the importance of providing default models whenever possible, but in this case, retraining was necessary to ensure a fair and accurate comparison on the same test set after employing the same training and validation sets.

2. The authors state on lines 506-507 in reference to Table 4, “we do not have the performance for all individual proteins for most methods.” Did the authors take the results on the test set from the papers directly? How do we know that the reported results are accurate? The authors should download each program and run them locally on their test set if possible to verify the performance of each method. This should be done for all results presented in the manuscript.

Reply: Thank you for the comments. We agree that it would be better if one could obtain pretrained models for all methods and perform the comparison. Unfortunately, although some provided source codes, none has provided pre-trained model weights. Thus, we simple quoted the results from the PiFold paper, where the comparison was made by re-training the methods. Because we have reproduced ProteinMPNN and PiFold results reimplemented in the PiFold paper, it is safe to quote all other results.

3. I would suggest that the authors add in a comparison with RosettaFixBB and/or RosettaFlexibleBB, which are probably two of the most widely-used energy function-based methods for protein sequence design.

Reply: Per suggestion, we now added the results from RosettaFixBB. The results are now reported in newly revised Tables 4, 5, and 6, Figures 2, and 4 and Supplementary Table S2. We also added RosettaFixBB in the Methods section and in the main text accompanying these figures and tables.

**Table 4.** Method comparison on the whole CATH4.2 test set according to perplexity and median native sequence recovery**.**

| Methods | Perplexity ↓ | | | Median Recovery (%) ↑ | | | |  |
| --- | --- | --- | --- | --- | --- | --- | --- | --- |
|  | Short^a^ | Single-chain^b^ | All | | Short^a^ | Single-chain^b^ | All | |
| Energy-based Protein Design | | | | | | | | |
| RosettaFixBB | - | - | - | | 18.49 | 19.05 | 31.18 | |
| OSCAR-design | - | - | - | | 23.18 | 21.48 | 34.83 | |
| AI-based Protein Design | | | | | | | | |
| StructGNN^c^ | 8.29 | 8.74 | 6.40 | | 29.44 | 28.26 | 35.91 | |
| GraphTrans^c^ | 8.39 | 8.83 | 6.63 | | 28.14 | 28.46 | 35.82 | |
| GCA^c^ | 7.09 | 7.49 | 6.05 | | 32.62 | 31.10 | 37.64 | |
| GVP^c^ | 7.23 | 7.84 | 5.36 | | 30.60 | 28.95 | 39.47 | |
| AlphaDesign^c^ | 7.32 | 7.63 | 6.30 | | 34.16 | 32.66 | 41.31 | |
| ProteinMPNN | | | | | | | | |
| - implemented by Gao et al^c^ | 6.21 | 6.68 | 4.61 | | 36.35 | 34.43 | 45.96 | |
| - implemented in this work | 6.28 | 6.71 | 4.68 | | 33.58 | 31.65 | 46.15 | |
| PiFold | | | | | | | | |
| - implemented by Gao et al^c^ | 6.04 | 6.31 | 4.55 | | 39.84 | 38.35 | 51.66 | |
| - implemented in this work | 5.93 | 6.15 | 4.59 | | 38.10 | 37.61 | 51.55 | |
| LM-DESIGN + PiFold^d^ | 6.77 | 6.46 | 4.52 | | 37.88 | 42.47 | **55.65** | |
| SPIN-CGNN | **5.01** | **5.02** | **4.05** | | **44.71** | **43.37** | 54.81 | |

^a^ Short: the sub test set for those targets with less than 100 amino acids.

^b^ Single-chain: the sub test set for those targets from single-chain structures.

^c^ Results adapted from Ref. ^25^.

^d^ Results adapted from Ref. ^26^.

**Table 5.** Comparison of sequences designed by SPIN-CGNN, RosettaFixBB, OSCAR-design, ProteinMPNN, and PiFold on CATH4.2-StructNR193 and PDB-StructNR156 test sets according to perplexity, median sequence recovery, median relative deviation of the frequency of amino-acid residue types, the median relative BLOSUM score, fraction of low complexity regions, conservation of hydrophobic and hydrophilic sequence positions, the mean steric clash count of refolded structures, and the difference between refolded and target structures in term of RMSD, GDT-TS and TM-score.

| Methods | | Perplexity ↓ | Median Recovery (%) ↑ | | AA Compositions  (Median Rel. Dev.) ↓ | | AA Substitutions  (Median Relative BLOSUM score)↑ | | LCR (%) ↓ | | Hydrophobic Conservation (%) ↑ | | AlphaFold2 Prediction Test | | | | |
| --- | --- | --- | --- | --- | --- | --- | --- | --- | --- | --- | --- | --- | --- | --- | --- | --- | --- |
|  |  |  |  |  |  |  |  |  |  |  |  |  | Median RMSD (Å) ↓ | Median GDT-TS ↑ | Median  TM-score ↑ | Mean Clash Count ↓ | |
| CATH4.2-StructNR193 | | | | | | | | | | | | | | | | |  |
| Native (Reference) | - | | | - | | - | | - | | 4.12 | | - | 1.85 | 83.82 | 0.863 | 0.021 | |
| RosettaFixBB | - | | | 29.29 | | 0.141 | | - | | 10.14 | | 68.16 | 5.01 | 52.91 | 0.759 | **0.053** | |
| OSCAR-design | - | | | 33.33 | | **0.078** | | - | | **8.35** | | 71.22 | 3.17 | 63.95 | 0.819 | 0.088 | |
| ProteinMPNN | 4.97 | | | 43.82 | | 0.357 | | 0.362 | | 23.95 | | 74.02 | 2.75 | 67.97 | 0.812 | 0.130 | |
| PiFold | 4.88 | | | 50.00 | | 0.145 | | 0.394 | | 13.31 | | 80.99 | 2.41 | 75.88 | 0.847 | 0.321 | |
| SPIN-CGNN | **4.36** | | | **52.50** | | 0.099 | | **0.442** | | 11.22 | | **82.50** | **2.09** | **79.13** | **0.857** | 0.078 | |
| PDB-StructNR156 | | | | | | | | | | | | | | | | |  |
| Native (Reference) | - | | | - | | - | | - | | 4.27 | | - | 1.89 | 83.68 | 0.903 | 0.944 | |
| RosettaFixBB | - | | | 34.78 | | 0.169 | | - | | 8.30 | | 70.40 | 2.58 | 72.09 | 0.869 | **0.879** | |
| OSCAR-design | - | | | 39.41 | | 0.067 | | - | | 7.80 | | 74.17 | 2.62 | 75.79 | 0.890 | 1.246 | |
| ProteinMPNN | 4.04 | | | 47.26 | | 0.306 | | 0.433 | | 12.93 | | 77.07 | 2.21 | 76.50 | 0.891 | 1.855 | |
| PiFold | 4.00 | | | 54.81 | | 0.110 | | 0.459 | | 6.56 | | 82.56 | 1.88 | 81.17 | 0.899 | 1.779 | |
| SPIN-CGNN | **3.42** | | | **58.88** | | **0.063** | | **0.517** | | **5.49** | | **85.44** | **1.75** | **85.65** | **0.913** | 1.101 | |

**Table 6.** Comparison of sequences designed by SPIN-CGNN, ProDesign-LE, RosettaFixBB, OSCAR-design, ProteinMPNN, and PiFold on Hallucination129 and Diffusion100 test sets according to fraction of low complexity regions, median relative deviation of the frequency of amino-acid residue types, the mean steric clash count of refolded structures, and the difference between refolded and target structures in term of RMSD, GDT-TS and TM-score.

| Methods | LCR (%) ↓ | AA Compositions  (Median Rel. Dev.) ↓^a^ | AlphaFold2 Prediction Test | | | |
| --- | --- | --- | --- | --- | --- | --- |
|  |  |  | Median RMSD (Å) ↓ | Median GDT-TS ↑ | Median TM-score ↑ | Mean Clash Count ↓ |
| Hallucination129 | | | | | | |
| ProDesign-LE^28^ | - | - | - | - | 0.83 (Mean) | - |
| RosettaFixBB | 19.94 | 0.412 | 1.55 | 83.50 | 0.901 | 0.016 |
| OSCAR-design | 11.0 | 0.390 | **1.10** | **91.25** | **0.935** | **0.000** |
| ProteinMPNN | 32.96 | 0.565 | 1.38 | 84.75 | 0.903 | 0.023 |
| PiFold | 17.19 | **0.289** | 1.26 | 88.25 | 0.916 | 0.078 |
| SPIN-CGNN | 14.91 | 0.387 | 1.19 | 89.00 | 0.923 | 0.039 |
| Diffusion100 | | | | | | |
| RosettaFixBB | 14.12 | 0.406 | 4.04 | 53.25 | 0.768 | **0.000** |
| OSCAR-design | **5.01** | **0.322** | 2.54 | 72.88 | 0.855 | 0.040 |
| ProteinMPNN | 56.32 | 0.518 | 3.47 | 59.00 | 0.767 | 0.080 |
| PiFold | 32.73 | 0.386 | 2.26 | 76.00 | **0.879** | 0.190 |
| SPIN-CGNN | 23.16 | 0.362 | **1.91** | **81.13** | 0.878 | 0.070 |

^a^ The average native amino acid frequency of the CATH4.2-StructNR193 and PDB-StructNR156 test set were taken as the reference compositions.

**Figure 2.** Deviation of the frequency of an amino acid in designed sequences from that in the native sequences by RosettaFixBB, OSCAR-design, ProteinMPNN, PiFold and SPIN-CGNN**.** (A) CATH4.2-StructNR193 and (B) PDB-StructNR156 test set.

**Figure 4.** Deviations of the structures of designed sequences predicted by AlphaFold2 from their respective target structures on four separate test sets from left to right panels (CATH4.2-StructNR193, PDB-StructNR156, Hallucination129, and Diffusion100 test set) evaluated according to RMSD (Å), GDT-TS, and TM-score (from top to bottom panels). The statistical significance of the difference of a given method to SPIN-CGNN was marked with ‘**’ for highly statistically significant (p-value<0.01), ‘*’ for statistically significant (0.01<p-value<0.05), and ‘-’ for not statistically significant (p-value>0.05). Specific p-values are presented in Supplementary Table S4.

4. What is the formula for calculating the LCR in Tables 5 and 6?

Reply: The LCRs in this study were detected by SEG algorithm. SEG identifies approximate segments of low complexity using a sliding window in the first pass and optimized these segments in the second pass. The optimized segments with the information measure lower than a given threshold will be marked as an LCR. Specifically, we run the SEG algorithm with the default setting from NCBI C++ toolkit, which is 12 for sliding window size and 2.2 for low-information cutoff. We now provided the description of LCR calculation as below:

“Thus, we also examined low complexity regions, which is the subsequences that normally lead to intrinsically disordered regions and the inability to fold into the target structure. We detected these subsequences using the SEG algorithm [45]. SEG identifies approximate segments of low complexity using a sliding window in the first pass and optimized these segments in the second pass. The optimized segments with the information measure lower than a given threshold will be marked as an LCR. Specifically, we run the SEG algorithm with the default setting from NCBI C++ toolkit, which is 12 for sliding window size and 2.2 for low-information cutoff. The fractions of low complexity regions were calculated as a criterion for analysis, denoted as LCR.”

Reference

[45] Wootton J C, Federhen S. Statistics of local complexity in amino acid sequences and sequence databases. Computers & chemistry, 1993, 17(2): 149-163.

5. In Table 5, what is the sequence recovery rate in buried vs solvent exposed regions of the protein. Are the deep learning methods very good at recovering buried regions but not at recovering exposed regions? Do the physical energy-based methods outperform the deep learning methods on the exposed regions?

Reply: We reported the dependence of sequence recovery on solvent accessibility in Supplementary Figure S5. We now provided an expanded report on these results. Deep-learning-based methods consistently outperform energy-based techniques (OSCAR-design and RosettaFixBB) in both core and surface regions. In the manuscript, we have added:

“Moreover, Deep-learning-based methods consistently outperform energy-based techniques (OSCAR-design and RosettaFixBB) in both core and surface regions.”

**Supplementary Figure S5.** The median sequence recovery of protein targets as a function of the fraction of surface residues on CATH4.2-StructNR193 (A) and PDB-Struct156 (B) test sets given by SPIN-CGNN, in comparison with a number of other methods as labeled. Nearly identical dependence on fraction of surface residues by SPIN-CGNN for two different test sets indicates the robustness of the methods for different datasets.

6. In Table 5, what is the BLOSUM score for OSCAR-design. Currently, the BLOSUM score is calculated based on the probability distribution similar to the perplexity, making it impossible to calculate this value for OSCAR-design. However, the authors may additionally calculate the BLOSUM score based on the actual designed sequences similar to the sequence recovery rate. Do the physics-based methods have more favorable BLOSUM scores than the deep learning methods? And if so, what does this mean?

Reply: This is a good question. However, calculating the BLOSUM score required the probability for residues at each position, which OSCAR-design does not provide. In principle, we could design 100 sequences for each protein and compute probabilities. However, it would take several dedicated months for CPU times with our current computing resource for 350 test proteins (350x100=35,000 designs). This computational requirement is prohibitive for our available computing resource. This is now clarified in the paper.

“It should be mentioned here that we did not calculate BLOSUM score for OSCAR-design and RosettaFixBB because it does not yield the probability of amino acid residues in one design as in AI-based methods. Although in principle one could perform 100 designs for each protein to obtain the probability, computational requirement is prohibitive for our available computing resource when applying to the large dataset we are employing here for test.”

7. In Table 6, what is the sequence recovery rate and AA composition deviation for each of the methods on the Hallucination129 test set?

Reply: The native sequence recovery and the AA composition deviation for designed sequences are not available since the hallucinated structures are generated structures without native sequences. This is now clarified in the paper.

“The Hallucination129 test set is made of the structures generated by hallucination and, thus, each structure does not have a native sequence to calculate sequence recovery. The same is true for the Diffusion100 set.”

8. In Tables 5 and 6, how was AlphaFold2 run? Were just the single designed sequences used or did the authors use MSAs? Also, were templates with >30% sequence identity to the queries excluded. If not, the TM-scores may be better for the deep learning methods as the native structures themselves could be identified as templates given the high sequence identity between the native and designed sequences.

Reply: As with previous studies (for example, ProDesign-LE), we run AlphaFold2 with default setting, including the default MSA and template searching. To address the comment regarding the potential use of homologous templates, we rerun AlphaFold2 by turning off template search. Results show that the refoldability of all methods tested without templates are slightly worse in the new results on the test sets with native structures (CATH.4.2-StructNR193 and PDB-StructNR156). Interestingly, we found that the effects on SPIN-CGNN are the smallest, when compared to other methods. Refolding RMSD by SPIN-CGNN increases 0.57 or 0.38 Å only for CATH.4.2-StructNR193 and PDB-StructNR156 datasets, respectively, after turning off templates, compared to an increase of +0.88/+0.42 Å for PiFold, +0.81/+0.39 Å for ProteinMPNN, +0.95/+0.68 Å for OSCAR-design. Thus, sequences designed by SPIN-CGNN are less dependent on templates contained in AlphaFold2 refolding, when comparing to other methods.

For the datasets of artificially generated structures (Hallucination129 and Diffusion100), excluding templates barely changes the results as expected.

We have replaced the AlphaFold2 test results in Tables 5 and 6 and Figure 4 with results excluding templates. Previous results with templates on are now included in the supplementary data as Supplementary Table S2, and Supplementary Figure S9. The following is added to the context:

“We noted that including templates in AlphaFold2 prediction only leads to small improvement in refolded structures as shown in Supplementary Table S2, and Supplementary Figure S9.”

**Table 5.** Comparison of sequences designed by SPIN-CGNN, RosettaFixBB, OSCAR-design, ProteinMPNN, and PiFold on CATH4.2-StructNR193 and PDB-StructNR156 test sets according to perplexity, median sequence recovery, median relative deviation of the frequency of amino-acid residue types, the median relative BLOSUM score, fraction of low complexity regions, conservation of hydrophobic and hydrophilic sequence positions, the mean steric clash count of refolded structures, and the difference between refolded and target structures in term of RMSD, GDT-TS and TM-score.

| Methods | | Perplexity ↓ | Median Recovery (%) ↑ | | AA Compositions  (Median Rel. Dev.) ↓ | | AA Substitutions  (Median Relative BLOSUM score)↑ | | LCR (%) ↓ | | Hydrophobic Conservation (%) ↑ | | AlphaFold2 Prediction Test | | | | |
| --- | --- | --- | --- | --- | --- | --- | --- | --- | --- | --- | --- | --- | --- | --- | --- | --- | --- |
|  |  |  |  |  |  |  |  |  |  |  |  |  | Median RMSD (Å) ↓ | Median GDT-TS ↑ | Median  TM-score ↑ | Mean Clash Count ↓ | |
| CATH4.2-StructNR193 | | | | | | | | | | | | | | | | |  |
| Native (Reference) | - | | | - | | - | | - | | 4.12 | | - | 1.85 | 83.82 | 0.863 | 0.021 | |
| RosettaFixBB | - | | | 29.29 | | 0.141 | | - | | 10.14 | | 68.16 | 5.01 | 52.91 | 0.759 | **0.053** | |
| OSCAR-design | - | | | 33.33 | | **0.078** | | - | | **8.35** | | 71.22 | 3.17 | 63.95 | 0.819 | 0.088 | |
| ProteinMPNN | 4.97 | | | 43.82 | | 0.357 | | 0.362 | | 23.95 | | 74.02 | 2.75 | 67.97 | 0.812 | 0.130 | |
| PiFold | 4.88 | | | 50.00 | | 0.145 | | 0.394 | | 13.31 | | 80.99 | 2.41 | 75.88 | 0.847 | 0.321 | |
| SPIN-CGNN | **4.36** | | | **52.50** | | 0.099 | | **0.442** | | 11.22 | | **82.50** | **2.09** | **79.13** | **0.857** | 0.078 | |
| PDB-StructNR156 | | | | | | | | | | | | | | | | |  |
| Native (Reference) | - | | | - | | - | | - | | 4.27 | | - | 1.89 | 83.68 | 0.903 | 0.944 | |
| RosettaFixBB | - | | | 34.78 | | 0.169 | | - | | 8.30 | | 70.40 | 2.58 | 72.09 | 0.869 | **0.879** | |
| OSCAR-design | - | | | 39.41 | | 0.067 | | - | | 7.80 | | 74.17 | 2.62 | 75.79 | 0.890 | 1.246 | |
| ProteinMPNN | 4.04 | | | 47.26 | | 0.306 | | 0.433 | | 12.93 | | 77.07 | 2.21 | 76.50 | 0.891 | 1.855 | |
| PiFold | 4.00 | | | 54.81 | | 0.110 | | 0.459 | | 6.56 | | 82.56 | 1.88 | 81.17 | 0.899 | 1.779 | |
| SPIN-CGNN | **3.42** | | | **58.88** | | **0.063** | | **0.517** | | **5.49** | | **85.44** | **1.75** | **85.65** | **0.913** | 1.101 | |

**Table 6.** Comparison of sequences designed by SPIN-CGNN, ProDesign-LE, RosettaFixBB, OSCAR-design, ProteinMPNN, and PiFold on Hallucination129 and Diffusion100 test sets according to fraction of low complexity regions, median relative deviation of the frequency of amino-acid residue types, the mean steric clash count of refolded structures, and the difference between refolded and target structures in term of RMSD, GDT-TS and TM-score.

| Methods | LCR (%) ↓ | AA Compositions  (Median Rel. Dev.) ↓^a^ | AlphaFold2 Prediction Test | | | |
| --- | --- | --- | --- | --- | --- | --- |
|  |  |  | Median RMSD (Å) ↓ | Median GDT-TS ↑ | Median TM-score ↑ | Mean Clash Count ↓ |
| Hallucination129 | | | | | | |
| ProDesign-LE^28^ | - | - | - | - | 0.83 (Mean) | - |
| RosettaFixBB | 19.94 | 0.412 | 1.55 | 83.50 | 0.901 | 0.016 |
| OSCAR-design | 11.0 | 0.390 | **1.10** | **91.25** | **0.935** | **0.000** |
| ProteinMPNN | 32.96 | 0.565 | 1.38 | 84.75 | 0.903 | 0.023 |
| PiFold | 17.19 | **0.289** | 1.26 | 88.25 | 0.916 | 0.078 |
| SPIN-CGNN | 14.91 | 0.387 | 1.19 | 89.00 | 0.923 | 0.039 |
| Diffusion100 | | | | | | |
| RosettaFixBB | 14.12 | 0.406 | 4.04 | 53.25 | 0.768 | **0.000** |
| OSCAR-design | **5.01** | **0.322** | 2.54 | 72.88 | 0.855 | 0.040 |
| ProteinMPNN | 56.32 | 0.518 | 3.47 | 59.00 | 0.767 | 0.080 |
| PiFold | 32.73 | 0.386 | 2.26 | 76.00 | **0.879** | 0.190 |
| SPIN-CGNN | 23.16 | 0.362 | **1.91** | **81.13** | 0.878 | 0.070 |

^a^ The average native amino acid frequency of the CATH4.2-StructNR193 and PDB-StructNR156 test set were taken as the reference compositions.

**Figure 4.** Deviations of the structures of designed sequences predicted by AlphaFold2 from their respective target structures on four separate test sets from left to right panels (CATH4.2-StructNR193, PDB-StructNR156, Hallucination129, and Diffusion100 test set) evaluated according to RMSD (Å), GDT-TS, and TM-score (from top to bottom panels). The statistical significance of the difference of a given method to SPIN-CGNN was marked with ‘**’ for highly statistically significant (p-value<0.01), ‘*’ for statistically significant (0.01<p-value<0.05), and ‘-’ for not statistically significant (p-value>0.05). Specific p-values are presented in Supplementary Table S4.

9. The results in Tables 5 and 6 are very interesting. For example, the median sequence recovery rate for SPIN-CGNN is nearly 60% higher than the physical energy function-based method, OSCAR-design; however, OSCAR-design produces sequences with AA compositions closer to the native distributions. Does this mean that the OSCAR-design sequences are more physically realistic? Additionally, why are the results for the deep learning methods significantly better than the physical energy function-based methods on the native structures but worse on the designed structures? Are SPIN-CGNN and the other deep learning-based methods very good at recapitulating high-confidence, conserved regions in the protein structures but fail to recapitulate less conserved regions? What are the physical characteristics of the designs produced by the deep learning methods? Do they introduce steric clashes or physically unrealistic residues? I believe a closer examination of the physical quality and stability of the designs is warranted. The authors could run MD simulations to verify the stability of the designed sequences and provide a closer examination of the physical quality of the designs by examining such things as steric clashes and MolProbity scores, among others.

Reply: Thank the reviewer to raise this interesting question. Indeed, despite of significantly lower sequence similarity, OSCAR-design has an AA composition closer to native AA composition than SPIN-CGNN for the CATH4.2-StructNR193 dataset. It is quite possible that this is in part due to taking into account of physical side-chains in OSCAR-design. However, it does not necessary mean that OSCAR-design will have a higher success rate than SPIN-CGNN in protein design because AA composition is only one of many measures. For example, for the same test set, SPIN-CGNN has a much higher conservation of hydrophobic positions than OSCAR-design (82.5% versus 71.2%). Conservation of hydrophobic positions is essential for a stable, soluble protein that requires a hydrophobic core and a hydrophilic surface. Thus, the only way to be sure about the performance of a method is to conduct wet lab experiments, which is the subject of our future work.

We are unable to perform MD simulations to examine the stabilities of designed proteins because the computational resources for simulating hundreds of proteins for hundreds of nanoseconds are not available (some proteins are very large). Per suggestion, we now investigated the atomic clashes by examining the atomic structures refolded by AlphaFold2 which has internal minimization steps to remove atomic clashes. Atomic clashes in AlphaFold2-refolded structures for all methods are low for all designed proteins (Tables 5 and 6). Physical-based methods have lower atomic clashes due to their built-in avoidance of atomic clashes.

Tables 5 and 6 indicate that the deep learning methods significantly better than the physical energy function-based methods when designing on the native structures but worse on the generated structures in term of low complexity regions. We believe that this is because “generated” structures from diffusion models, in particular, do not explicitly consider specific sidechains. As a result, the geometrical characters of main chains and the physical space between mainchain atoms in these designed structures maybe more favorable for most popular residue types with small sizes. These structures would be prone to produce sequences with low complexity, regardless of physical or deep-learning based methods as shown. The following was added in the text.

“Table 6 shows that although most methods can refold designed sequences to the corresponding generated structures well, both physical and AI-based methods have elevated factions of low-complexity regions and larger deviation of amino acid compositions from natural proteins. This may be because “generated” structures, particularly from diffusion models, do not explicitly consider specific sidechains. As a result, the geometrical characters of main chains and the physical space between mainchain atoms in these designed structures maybe more favorable for most popular residue types (as shown in Figure 2). These structures would be prone to produce sequences with low complexity, regardless of physical or deep-learning based methods.”

**Table 5.** Comparison of sequences designed by SPIN-CGNN, RosettaFixBB, OSCAR-design, ProteinMPNN, and PiFold on CATH4.2-StructNR193 and PDB-StructNR156 test sets according to perplexity, median sequence recovery, median relative deviation of the frequency of amino-acid residue types, the median relative BLOSUM score, fraction of low complexity regions, conservation of hydrophobic and hydrophilic sequence positions, the mean steric clash count of refolded structures, and the difference between refolded and target structures in term of RMSD, GDT-TS and TM-score.

| Methods | | Perplexity ↓ | Median Recovery (%) ↑ | | AA Compositions  (Median Rel. Dev.) ↓ | | AA Substitutions  (Median Relative BLOSUM score)↑ | | LCR (%) ↓ | | Hydrophobic Conservation (%) ↑ | | AlphaFold2 Prediction Test | | | | |
| --- | --- | --- | --- | --- | --- | --- | --- | --- | --- | --- | --- | --- | --- | --- | --- | --- | --- |
|  |  |  |  |  |  |  |  |  |  |  |  |  | Median RMSD (Å) ↓ | Median GDT-TS ↑ | Median  TM-score ↑ | Mean Clash Count ↓ | |
| CATH4.2-StructNR193 | | | | | | | | | | | | | | | | |  |
| Native (Reference) | - | | | - | | - | | - | | 4.12 | | - | 1.85 | 83.82 | 0.863 | 0.021 | |
| RosettaFixBB | - | | | 29.29 | | 0.141 | | - | | 10.14 | | 68.16 | 5.01 | 52.91 | 0.759 | **0.053** | |
| OSCAR-design | - | | | 33.33 | | **0.078** | | - | | **8.35** | | 71.22 | 3.17 | 63.95 | 0.819 | 0.088 | |
| ProteinMPNN | 4.97 | | | 43.82 | | 0.357 | | 0.362 | | 23.95 | | 74.02 | 2.75 | 67.97 | 0.812 | 0.130 | |
| PiFold | 4.88 | | | 50.00 | | 0.145 | | 0.394 | | 13.31 | | 80.99 | 2.41 | 75.88 | 0.847 | 0.321 | |
| SPIN-CGNN | **4.36** | | | **52.50** | | 0.099 | | **0.442** | | 11.22 | | **82.50** | **2.09** | **79.13** | **0.857** | 0.078 | |
| PDB-StructNR156 | | | | | | | | | | | | | | | | |  |
| Native (Reference) | - | | | - | | - | | - | | 4.27 | | - | 1.64 | 86.93 | 0.908 | 1.026 | |
| RosettaFixBB | - | | | 34.78 | | 0.169 | | - | | 8.30 | | 70.40 | 2.27 | 75.83 | 0.892 | 0.605 | |
| OSCAR-design | - | | | 39.41 | | 0.067 | | - | | 7.80 | | 74.17 | 1.94 | 83.89 | 0.903 | 0.865 | |
| ProteinMPNN | 4.04 | | | 47.26 | | 0.306 | | 0.433 | | 12.93 | | 77.07 | 1.82 | 82.08 | 0.908 | 1.051 | |
| PiFold | 4.00 | | | 54.81 | | 0.110 | | 0.459 | | 6.56 | | 82.56 | 1.46 | 87.48 | 0.920 | 0.814 | |
| SPIN-CGNN | **3.42** | | | **58.88** | | **0.063** | | **0.517** | | **5.49** | | **85.44** | **1.37** | **90.45** | **0.924** | **0.603** | |

**Table 6.** Comparison of sequences designed by SPIN-CGNN, ProDesign-LE, RosettaFixBB, OSCAR-design, ProteinMPNN, and PiFold on Hallucination129 and Diffusion100 test sets according to fraction of low complexity regions, median relative deviation of the frequency of amino-acid residue types, the mean steric clash count of refolded structures, and the difference between refolded and target structures in term of RMSD, GDT-TS and TM-score.

| Methods | LCR (%) ↓ | AA Compositions  (Median Rel. Dev.) ↓^a^ | AlphaFold2 Prediction Test | | | |
| --- | --- | --- | --- | --- | --- | --- |
|  |  |  | Median RMSD (Å) ↓ | Median GDT-TS ↑ | Median TM-score ↑ | Mean Clash Count ↓ |
| Hallucination129 | | | | | | |
| ProDesign-LE^28^ | - | - | - | - | 0.83 (Mean) | - |
| RosettaFixBB | 19.94 | 0.412 | 1.55 | 83.50 | 0.901 | 0.016 |
| OSCAR-design | 11.0 | 0.390 | **1.10** | **91.25** | **0.935** | **0.000** |
| ProteinMPNN | 32.96 | 0.565 | 1.38 | 84.75 | 0.903 | 0.023 |
| PiFold | 17.19 | **0.289** | 1.26 | 88.25 | 0.916 | 0.078 |
| SPIN-CGNN | 14.91 | 0.387 | 1.19 | 89.00 | 0.923 | 0.039 |
| Diffusion100 | | | | | | |
| RosettaFixBB | 14.12 | 0.406 | 4.04 | 53.25 | 0.768 | **0.000** |
| OSCAR-design | **5.01** | **0.322** | 2.54 | 72.88 | 0.855 | 0.040 |
| ProteinMPNN | 56.32 | 0.518 | 3.47 | 59.00 | 0.767 | 0.080 |
| PiFold | 32.73 | 0.386 | 2.26 | 76.00 | **0.879** | 0.190 |
| SPIN-CGNN | 23.16 | 0.362 | **1.91** | **81.13** | 0.878 | 0.070 |

^a^ The average native amino acid frequency of the CATH4.2-StructNR193 and PDB-StructNR156 test set were taken as the reference compositions.

Reviewer 2:

1. In lines 200-202, the u_i and v_i should be defined in line 192.

Reply: Thank you. We now added these definitions.

2. Lines 423-424, “ProteinMPNN reimplemented model” is somewhat confusing. I found that ProteinMPP was also reimplemented in ref. 25 and the rate of 45.96% was from the reproduced version therein. Thus, I suggest the authors change “ProteinMPNN reimplemented model” to “ProteinMPNN implemented by Gao et al.”

Reply: Thank you. Modification has been made as suggested.

3. In Table 2, “4.36±0.01” in the “Model 3” row should also be highlighed.

Reply: Correction has been made as mentioned.

4. In Table 4, SPIN-CGNN outperformed “LM-DESIGN + PiFold” all categories of perplexity test and the “Short” and “Single-chain” categories of Median recovery (~7% and ~1%, respectively) but was ~1% worse in “All” of Median recovery. Could the authors compare the performance of SPIN-CGNN and “LM-DESIGN + PiFold” on the targets that are not categorized as “Short” or “Single-chain”, so that readers can understand how much worse SPIN-CGNN on these targets?

Reply: This is a good question. The results listed in Table 4 about LM-DESIGN+PiFold were obtained from their paper. We did not have the specific recovery of each test target or an official-released model of LM-DESIGN+PiFold for reproducing these data. As a result, we are unable to check on details.

5. The concept of “low complexity region” should be clearly defined in the manuscript. My understanding is the “loop” or “coil” region of a protein, but it should be clearly defined.

Reply: We now added the definition of LCRs as below.

“Thus, we also examined low complexity regions, which is the subsequences that normally lead to intrinsically disordered regions and the inability to fold into the target structure. We detected these subsequences using the SEG algorithm [45]. SEG identifies approximate segments of low complexity using a sliding window in the first pass and optimized these segments in the second pass. The optimized segments with the information measure lower than a given threshold will be marked as an LCR. Specifically, we run the SEG algorithm with the default setting from NCBI C++ toolkit, which is 12 for sliding window size and 2.2 for low-information cutoff. The fractions of low complexity regions were calculated as a criterion for analysis, denoted as LCR.”

6. The performance varies a lot between two test sets in Table 5. Instead of just listing the results there, I think it would be more interesting to explain these differences in greater detail. Could the authors add the LCR (%) for native sequences in this table as a reference? Nevertheless, is LCR (%) a good metric for assessing the design quality as they showed the AlphaFold2 prediction test? I also so because in CATH4.2-StructNR193, the LCR by OSCAR-design was the lowest, but its corresponding AlphaFold2 prediction test was the worst (in terms of RMSD and GDT-TS)? If we trust the global structure prediction metric, it seems that a lower LCR does not necessarily lead to a higher refolding ability.

Reply: The performance gap between two test sets can be explained by the difference between two test sets on the faction of surface residues, as discussed in Lines 532-540. The LCRs of native sequences are now added in Table 5. The disagreement between LCR and AlphaFold2 refolding does not necessary disqualify LCR as a metric. This is because refolding results by AlphaFold2 were produced by inputting designed sequences along with naturally occurring homologous sequences found by homolog search. The defects of LCR regions in the designed regions may be masked by lack of those LCR regions in naturally occurring homologous sequences. Higher sequence identities to native sequences by deep-learning-based methods over OSCAR-design will certainly lead to better refolding results due to improved homology search. Thus, we reported multiple measures in this paper for examining the quality of a designed sequence.

“We would like to emphasize the importance of using different measures to computationally assess the designed sequences. This is because native sequence recovery and the deviation from the native sequence (perplexity) only reflect one aspect of designed sequences. High sequence identity does not assure foldability as a few mutations are often found sufficient to disrupt structural stability. Moreover, too many hydrophobic residues on the protein surface will lead to insoluble and aggregated proteins and low complexity in sequences often leads to intrinsically disordered regions. In addition, the refoldability by AlphaFold2 is not that sensitive to the sequences given by different methods because designed sequences are now highly similar to the wild type sequences and, thus, naturally occurring homologous sequences were employed as a part of the input for AlphaFold2 (Tables 5 and 6). Here we showed that the fraction of low complexity regions remains much higher than native sequences and energy-based techniques, indicating the room for further improvement.”

7. In lines 693-694, “This new training set, however, led to a poorer performance for those unseen structures.” And lines 698-699 “we employed the whole training set as it improves the generalizability over the smaller training set.” Could the authors explain more reason on the poorer performance on the nonredundant, smaller training set. Is this because the “TMscore<0.4” is too strict, removing many nonredundant folds? Or is because TM-score is just a global metric and the larger set can keep more abundant local structural information which are very useful for training, and TM-score<0.4 will eliminate many useful local information?

Reply: Thank you for the question. We agree that the worse performance by using TM-score <0.4 is not only because “TMscore<0.4” is too strict, removing many nonredundant folds but also because TM-score is just a global metric and the larger set can keep more abundant local structural information which are very useful for training. We now have modified the discussion to reflect your excellent point. Unfortunately, it will be difficult to separate the contributions from these two different effects. Here is the added content in the discussion:

“The worse performance by using TM-score <0.4 is not only because it is too strict for removing many nonredundant folds but also because TM-score is just a global metric and the larger set can keep more abundant local structural information which are very useful for training. In this case, it will be difficult to separate the contributions from these two different effects. Thus, we employed the whole training set as it improves the generalizability over the smaller training set.”

8. The authors have tons of data, but they did not dig them very deeply, nor the Discussion section was discussed in depth.

Reply: We had added more discussions in the revised manuscript. Thank you for your comments).

“It is well known that backbone bond angles and torsion angles may contain information biased toward certain amino acid residues. For example, the $N$-$C_{\alpha}$-$C$ angle is within [121, 126] for histidine and within [117, 122] for leucine. Unlike other residues, Gly does not any forbidden regions in the ϕ-ψ torsion angle space. This leads to the question if removing these angles will lead to a significant change in method performance. To address this question, we trained a SPIN-CGNN model with inter-atomic distances as the edge features only. We found that the recovery of the distance-only model on native structures drops only slightly from 52.5% to 51.21% on the CATH4.2-StructNR193 test set and from 58.8% to 57.36% on the PDB-StructNR156 test set. Thus, the angles only have a small impact on residue-type recovery.

Two sets of generated model protein structures were also employed for testing various protein design techniques. They are Hallucination and Diffusion sets. Table 6 shows that although most methods can refold designed sequences to the corresponding generated structures well, both physical and AI-based methods have elevated factions of low-complexity regions and larger deviation of amino acid compositions from natural proteins. This may be because “generated” structures, particularly from diffusion models, do not explicitly consider specific sidechains. As a result, the geometrical characters of main chains and the physical space between mainchain atoms in these designed structures maybe more favorable for most popular residue types (as shown in Figure 2). These structures would be prone to produce sequences with low complexity, regardless of physical or deep-learning based methods.”

Reviewer 3:

1. Presentation of the approach: In the manuscript, the authors described the node updating and edge updating only and did not mention the procedure for sequence generating, although they showed two blocks in Figure 1. The authors should describe the complete procedure of sequence design to make readers easy to follow.

Reply: Thank you. We now included the description of the complete procedure at the beginning of Methods section.

“Figure 1 shows the overall workflow of SPIN-CGNN. The backbone structure in SPIN-CGNN is first represented as a graph with a distance-based contact graph representation. It takes in the coordinates of backbone atoms including $N$, $C\alpha$, $C$, $O$, and transforms them as node features for residues and edge features for inter-residues relationship. Moreover, the connections in the graph including edges and second-order edges were recorded for the computation of neural networks. Then, the represented graph will be inputted into the SPIN-CGNN blocks to iteratively extract structural features. An MLP will be applied on the updated node features to predict the probability of 20 amino acids for each position. Finally, protein sequences can be sampled based on the predicted probabilities”

2. Dominating factors: the approach is a mixture of a large variety of ingredients, including using virtual atoms and bond angle/length as node features, using inter-atomic vectors and distances as edge features, and using SK mechanism to merge multiple features. The mixture of multiple ingredients is useful; however, this also brings a question: Which ingredient is the dominating one that determines residue type at a position?

Reply: We did analyze the contributions from various features. As reported in Results 3.1~3.3, the contact-based graph construction, additional edge information, and SK modules all contributed to the improvement in SPIN-CGNN with about 1% recovery on CATH4.2-StructNR193 test set and PDB-StructNR156 test set. The result indicates cumulative improvement from multiple changes.

3. Robustness of the approach: the authors used bond angles as node features, which carry information of residue type, for example, the N-C-CA angle is within [121, 126] for histidine and within [117, 122] for leucine. This fact raises a question: is bond angle the key feature for determining residue type? If yes, does this mean that this approach relies on accurate structure by X-ray crystallography? Is this the reason that the performance of the approach is not that good on hallucinated proteins as on CATH proteins? I also suggest the authors to examine the performance of SPIN-CGNN on the structures generated by diffusion model.

Reply: Thank you for this great question. To evaluate whether the specific node features, especially those strongly related to the residue types, affected the robustness of this method, we trained a SPIN-CGNN model with inter-atomic distances as the edge features only. We found that the recovery of the distance-only model on native structures drops only slightly from 52.5% to 51.21% on the CATH4.2-StructNR193 test set and from 58.8% to 57.36% on the PDB-StructNR156 test set. Thus, the angles only have a small impact on residue-type recovery.

In addition, we now have added a new test set from a diffusion model. We examined the performance of SPIN-CGNN on the structures generated by the diffusion model and found that SPIN-CGNN did well according to the refolding by AlphaFold2 with a median RMSD of 1.91Å from the target structure for the Diffusion set. Together with the similar performance on the Hallucination set (a median RMSD of 1.19Å from the target structure), SPIN-CGNN performs robustly for non-native sets. However, there is a common problem for all deep learning models: high fractions of low complexity regions. This problem is likely caused by the lack of explicit modeling of sidechains in deep learning models and nonnative-like backbone conformations generated from Hallucination and Diffusion that biased toward specific residues with abundant populations. This is now discussed in the discussion section. We have also added descriptions on the diffusion dataset obtained.

“It is well known that backbone bond angles and torsion angles may contain information biased toward certain amino acid residues. For example, the $N$-$C_{\alpha}$-$C$ angle is within [121, 126] for histidine and within [117, 122] for leucine. Unlike other residues, Gly does not any forbidden regions in the ϕ-ψ torsion angle space. This leads to the question if removing these angles will lead to a significant change in method performance. To address this question, we trained a SPIN-CGNN model with inter-atomic distances as the edge features only. We found that the recovery of the distance-only model on native structures drops only slightly from 52.5% to 51.21% on the CATH4.2-StructNR193 test set and from 58.8% to 57.36% on the PDB-StructNR156 test set. Thus, the angles only have a small impact on residue-type recovery.”

“Two sets of generated model protein structures were also employed for testing various protein design techniques. They are Hallucination and Diffusion sets. Table 6 shows that although most methods can refold designed sequences to the corresponding generated structures well, both physical and AI-based methods have elevated factions of low-complexity regions and larger deviation of amino acid compositions from natural proteins. This may be because “generated” structures, particularly from diffusion models, do not explicitly consider specific sidechains. As a result, the geometrical characters of main chains and the physical space between mainchain atoms in these designed structures maybe more favorable for most popular residue types (as shown in Figure 2). These structures would be prone to produce sequences with low complexity, regardless of physical or deep-learning based methods.”

“For diffusion models, we constructed a new ‘Diffusion100’ test set by: a) generated 1000 structures with SE(3) diffusion model [36]; b) test the refoldability with the process from the paper of SE(3)-diffusion model. Specifically, we designed 8 sequences for each generated structure with ProteinMPNN, predicted structures for each sequence with ESMFold [37], calculated the median refold RMSD of 8 designed sequences; c) picked the 100 generated structures with the smallest median refold RMSD.”

Reference

[36] Yim J, Trippe B L, De Bortoli V, et al. SE(3) diffusion model with application to protein backbone generation. arXiv preprint arXiv:2302.02277, 2023.

[37] Lin Z, Akin H, Rao R, et al. Evolutionary-scale prediction of atomic-level protein structure with a language model. Science, 2023, 379(6637): 1123-1130.

4. Redundancy of node features: In addition, the authors used bond lengths as node features; however, the bond length is roughly the same for all residues. The effects of virtual atoms is also not clearly stated. Are these features really inforamtive for residue type determination?

Reply: We did not analyze these specific contributions because we followed PiFold’s lead on these features and the analysis was done in the paper. In the PiFold paper, after removing bond lengths from node features, the recovery on the test set decreased from 51.66% to 51.49%. By removing the number of virtual atoms from 3, to 2, 1, 0, the recovery gradually decreased from 51.66% to 51.51%, 51.10%, and 51.02%. Thus, these features made some useful contribution and we simply followed their lead on these features.

5. Organization of the Result section: I appreciate the author’s viewpoints that, for protein sequence design, the quality of the predicted structure is much more important than sequence recovery and perplexity. The current form of the manuscript puts “impact of graph construction” and two ablation studies as the first three results. I suggest the authors to reorganize the Result section with the quality of the predicted structures (subsection 3.11) as the first subsection. I also suggest the authors to add a column into Table 1, 2, 3, 4 to show the quality the predicted structures.

Reply: Thank you for the suggestion. The results were presented to illustrate how our method was optimized by changing graph construction (CGraph), message passing (SOE/sym), and finally message merging (SK). This optimization was done by using fast-computing properties of sequence recovery and perplexity. Only after optimization, we tested the final model by AlphaFold2, which is computationally intensive. The computing requirement is what prohibits us to use AlphaFold2 in model optimization.

6. Inconsistent figures: In both Table 2 and 3, the mean perplexity and recovery of SPINCGNN on CATH4.2 is 4.36 and 52.89, respectively. However, these figures are 4.05 and 54.81 in Table 4. Why? In addition, the head of Table 1, 2, 3 is “mean recovery” but the authors listed them in the form of standard variation.

Reply: The reason to the different numbers in Table 4 and Table 2/Table 3 is due to different test sets. The CATH4.2-StructNR193 test set in Table 2 and 3 is a non-redundance subset containing 193 targets from the whole CATH4.2 test set in Table 4, which containing 1120 targets.

For the ablation studies presented in Tables 1, 2, and 3, we run 5 parallel training for each model or graph construction setting, and compare the average performance over 5 parallel training. The standard deviations of the performance of 5 parallel test are denoted so that we can determined whether the impact of modules or graph construction are statistically significant. This is now noted in Table footnotes.

7. The effect of SK: The SK technique calculates the sum of multiple features (Line 334) and then applies the multi-head mechanism on it. However, the simple addition of the features, say symmetric edge features, SOE and the extracted features (Line 281), might make one type of the features dominate the others. How about apply MLP on the concatenation of type of the features?

Reply: Before taking average or inputting into SK modules, all types of features had been normalized to prevent numerical domination. After the scaling in normalization, these features are not likely to dominate the others in the summation. We also run the experiments for ablation test of SK modules by replacing SK modules with two-layer MLP modules, per your suggestion. As shown in Supplementary Table S1, SPIN-CGNN continues to improve over than models without SK modules.

“We also evaluated the effect of SK with comparison to MLP modules (Supplementary Table S1) and observed similar improvement of using SK.”

**Supplementary Table S1.** Impact of the use of selective kernels in node update and edge update according to perplexity and median sequence recovery for two test datasets (CATH4.2-StructNR193 and PDB-StructNR156). Two-layer MLP modules were applied to replace SK modules for no-SK models.

| Model | Node SK | Edge SK | Perplexity ↓ ^a^ | Median  Recovery (%) ↑ ^a^ |
| --- | --- | --- | --- | --- |
| CATH4.2-StructNR193 | | | | |
| Model 7 | **×** | **×** | 4.49 ± 0.01 | 52.04 ± 0.33 |
| Model 8 | √ | **×** | 4.43 ± 0.02 | 52.43 ± 0.38 |
| Model 9 | **×** | √ | 4.47 ± 0.02 | 52.09 ± 0.41 |
| SPIN-CGNN | √ | √ | **4.36 ± 0.01** | **52.89 ± 0.64** |
| PDB-StructNR156 | | | | |
| Model 7 | **×** | **×** | 3.57 ± 0.00 | 57.08 ± 0.18 |
| Model 8 | √ | **×** | 3.53 ± 0.03 | 57.96 ± 0.26 |
| Model 9 | **×** | √ | 3.56 ± 0.02 | 57.61 ± 0.15 |
| SPIN-CGNN | √ | √ | **3.43 ± 0.02** | **58.54 ± 0.27** |

^a^ The average of 5 parallel tests with the standard deviations.

8. The effect of SOE: The message-passing mechanism aims to broadcast the information of the neighbors to the central residue even the neighbors are 1, 2, 3, … hops apart and thus naturally considers the information of secondary-order neighbors. The authors should perform case study to clearly show the effects of SOE updating.

Reply: This is a good question. We have performed the ablation study to demonstrate its usefulness (Table 2, Model 1 versus Model 3). The improvement is significant but small (~1% improvement in sequence recovery). An effect like this is difficult to illustrate by a case study. After a few trials, we decided not to present a case study for this purpose. This frustration also highlights the black box nature of deep learning.

9. Case study: I suggest the authors to provide case study to clearly state the most effective ingredients of the approach.

Reply: A case study is now added as shown in Figure 5, along with section 3.12 to show how the CGraph representation improved the fixed backbone design with an adaptive number of neighbors.

**“3.12 Case study**

To further understand how SPIN-CGNN improved fixed backbone protein design with contact graph (CGraph), we presented a case in Figure 5, where the structure of NADP-reducing hydrogenase subunit HndA (PDB 2AUV Chain A) was employed for protein design. SPIN-CGNN outperformed PiFold with a higher recovery (30.59% vs. 28.24%) and a much smaller refold RMSD (5.66 Å vs. 14.02 Å). The CGraph12 graph construction method captured more information from the compact core, as shown in Figure 5A with 47 neighboring residues for residue 10, compared to a fixed number of 30, when KNN-30 were employed.”

**Figure 5.** An illustrative example to highlight the dense local contacts accounted by SPIN-CGNN for improving fixed backbone design. (A) The neighbors for residue 10 (yellow) in PDB 2AUV chain A, which has the greatest number of neighbors determined by CGraph12 (magenta). (B) The AlphaFold2-predicted structure of sequence designed by SPIN-CGNN (magenta), aligned on the native PDB structure (green). (C) The neighbors determined by KNN-30 (cyan) for the same protein. (D) The corresponding AlphaFold2-predicted structure of sequence designed by PiFold (cyan).
