# Supplementary material for: SPIN-CGNN: Improved fixed backbone protein design with contact map-based graph construction and contact graph neural network
Source: PLoS Comput Biol. 2023 Dec 7;19(12):e1011330. doi: 10.1371/journal.pcbi.1011330 (PMC10729952; doi:10.1371/journal.pcbi.1011330)
Supplement: Attachment — Submitted filename: Response.docx [file pcbi.1011330.s016.docx]

From editor:

Based on the reviews, your manuscript may become acceptable, providing that you modify the manuscript according to the recommendations of the third reviewer. Meanwhile, please provide appropriate references to TM-score and GDT-score which have been extensively used in the manuscript but are not necessarily known to all readers. Meanwhile, necessary references are needed for justifying some of the specific score cutoffs (e.g., TM-score>0.4).

Reply: We now have added these two references on TM-score and GDT-score. Sorry for this unintentional omission. The use of TM-score>0.4 is based on the original paper which developed TM-Score, where it states that “the majority of the ‘Easy’ targets have TM-scores above 0.4, while most ‘Medium/Hard’ targets have TM-scores below 0.4, with an obvious gap between the ‘Easy’ and the ‘Medium/Hard’ target distributions”. Typically, two structures with TM-score >0.5 is considered as structures within the same structural fold. Using TM-score<0.4 is considered as a safe removal of structurally similar proteins. The original TM-score reference is now cited.

Reference:

35. Y. Zhang, J. Skolnick, Scoring function for automated assessment of protein structure template quality, Proteins, 2004, 57: 702-710.

49. Zemla A, Venclovas Č, Moult J, et al. Processing and analysis of CASP3 protein structure predictions. Proteins: Structure, Function, and Bioinformatics, 1999, 37(S3): 22-29.

Response to reviewers’ comments

Reviewer 3:

1. Dominating factors: the approach is a mixture of a large variety of ingredients and the authors’s analysis suggest that none of the ingredients is dominant and the power of the approach is essentially a “cumulative improvement from multiple changes”. This observation and conclusion are instructive and I suggest to add them to the Conclusion section.

Reply: We added this conclusion into Discussion section now.

“We proposed SPIN-CGNN, a deep learning-based method for fixed backbone protein design. Our approach incorporates several key elements, including contact-map graph construction, second-order edge updates, and selective kernels. Ablation studies reveal that the power of SPIN-CGNN lies in the cumulative improvement resulting from these multiple changes. Comparing our method with the most recently developed deep learning-based approaches, ProteinMPNN and PiFold, we found that SPIN-CGNN consistently outperformed them across various metrics including perplexity, sequence recovery, amino-acid composition, amino-acid substitution, low complexity regions, and conservations of hydrophobic/hydrophilic positions. Refolding of designed sequences by AlphaFold2 indicates that the structures produced by SPIN-CGNN are comparable to those by PiFold but significantly closer to target structures than ProteinMPNN.”

2. Redundancy of node features: the authors used bond lengths as node features; however, the bond length is roughly the same for all residues. The effects of virtual atoms is also not clearly stated. The authors cited PiFold’s analysis to answer this question, which shows only marginal contributions by these two types of features. I suggest to add a paragraph to clearly state the effects of them.

Reply: We now added a paragraph in the Methods Graph Representation section to state the components of PiFold features and its effects.

“We constructed the node features and edge features as in PiFold [25]. In addition to conventional features widely employed such as distance and direction between backbone atoms, the PiFold features further incorporated bond lengths, bond angles, and learnable virtual atoms. A series of ablation studies conducted to discern the individual contributions of these components revealed a slight enhancement in the recovery performance of PiFold in fixed backbone design.”

3. Writing: The current form of the manuscript still lacks of careful proofreading. There are still many grammar errors or mistakes., e.g.

— Supplementary Table S1. …. Two-layer MLP modules were applied to replace SK modules for no-SK models

— Ref. 35 even has Chinese characters.

— Ref. 39, 44: The major words of journal/conference names are not capitalized.

Reply: Thank you for pointing out the grammar mistakes. Corrections have been made in the new manuscript.

a) “Supplementary Table S1. …. Two-layer MLP modules were employed to substitute SK modules in models without selective kernels (no-SK models).”
b) “35. Zhihang C, Menglin J, Yifei Q. Research progress of artificial intelligence in designing protein structures. Synthetic Biology Journal, 2023, 4(3): 464-487.”
c) “39. Li X, Wang W, Hu X, et al. Selective kernel networks. Proceedings of the IEEE/CVF Conference on Computer Vision and Pattern Recognition. 2019: 510-519.

44. Tien M Z, Meyer A G, Sydykova D K, et al. Maximum allowed solvent accessibilites of residues in proteins. PloS One, 2013, 8(11): e80635.”
